# Postpartum Sacral Stress Fracture: An Atypical Case Report

**DOI:** 10.1155/2015/704393

**Published:** 2015-07-12

**Authors:** Andrea Speziali, Matteo Maria Tei, Giacomo Placella, Marco Chillemi, Giuliano Cerulli

**Affiliations:** ^1^Institute of Orthopedic and Traumatology, “Agostino Gemelli” Hospital, Catholic University, Largo Gemelli 8, 00168 Rome, Italy; ^2^Institute of Translational Research for Musculoskeletal System “Nicola Cerulli”, Via Einstein 12, 52100 Arezzo, Italy

## Abstract

Sacral stress fractures are common in elderly people. However, sacral stress fracture should be always screened in the differential diagnoses of low back pain during the postpartum period. We present a case of sacral fracture in a thirty-six-year-old woman with low back pain and severe right buttock pain two days after cesarean section delivery of a 3.9 Kg baby. The diagnosis was confirmed by MRI and CT scan, while X-ray was unable to detect the fracture. Contribution of mechanical factors during the cesarean section is not a reasonable cause of sacral fracture. Pregnancy and lactation could be risk factors for sacral stress fracture even in atraumatic delivery such as cesarean section. Our patient had no risk factors for osteoporosis except for pregnancy and lactation. Transient or focal osteoporosis is challenging to assess and it cannot be ruled out even if serum test and mineral density are within the normal range.

## 1. Introduction

Sacral stress fractures are rare, but they are a more common finding in athletes, such as long-distance runners, who report low back pain [1]. Sacral stress fractures are common in elderly people affected by osteoporosis or other morbidities such as rheumatoid arthritis, Paget disease, osteogenesis imperfecta, metastasis, and hyperparathyroidism or treated with radiation therapy [2].

However, sacral stress fracture should be always screened in the differential diagnosis of low back pain during the postpartum period.

The bones are subjected to changes during pregnancy, delivery, and postpartum period; thus hormonal and mechanical stresses influence the female body. Both hormones and mechanical loads can contribute to pathological conditions such as low back pain and buttock pain [3].

We report a case of a postdelivery sacral fracture.

## 2. Case Presentation

A thirty-six-year-old woman presented with low back pain and severe right buttock pain two days after cesarean section delivery of a 3.9 Kg baby. The patient was unable to sustain any weight bearing with the right limb. No findings of neurological abnormalities were found during the clinical investigation. The patient reported tenderness over right paraspinal muscle and over the right superior gluteal region and pain during deep palpation at right sacral and iliac compression. The patient had an antalgic gait pattern and evident limping.

She referred 14 kg weight gain during pregnancy and she did not show any symptoms of low back pain before the delivery. The cesarean section was previously decided due to the wrong position of the fetus and it was not done for an unsuccessful trial of convenient delivery.

**Figure 1:**
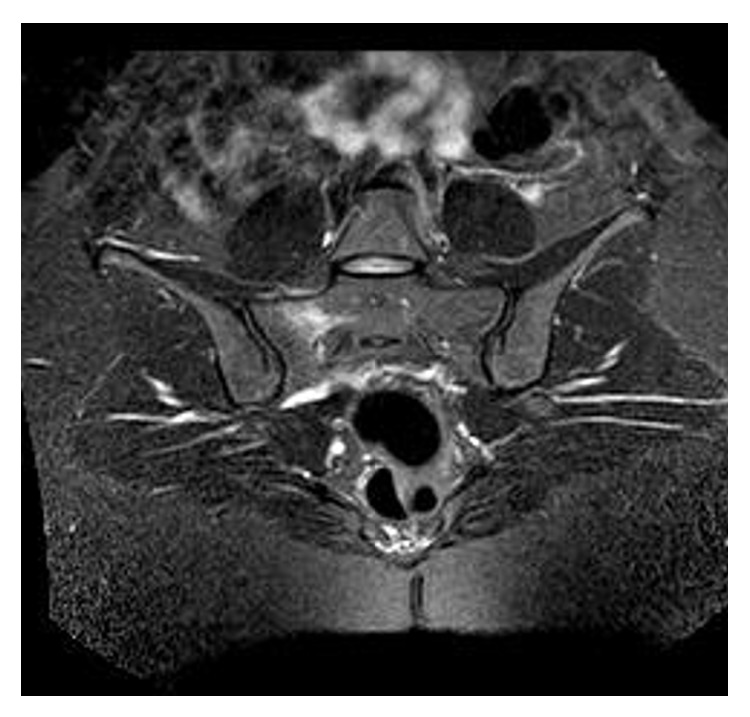
MRI T2 axial scan showed bone marrow edema at the sacrum.

**Figure 2:**
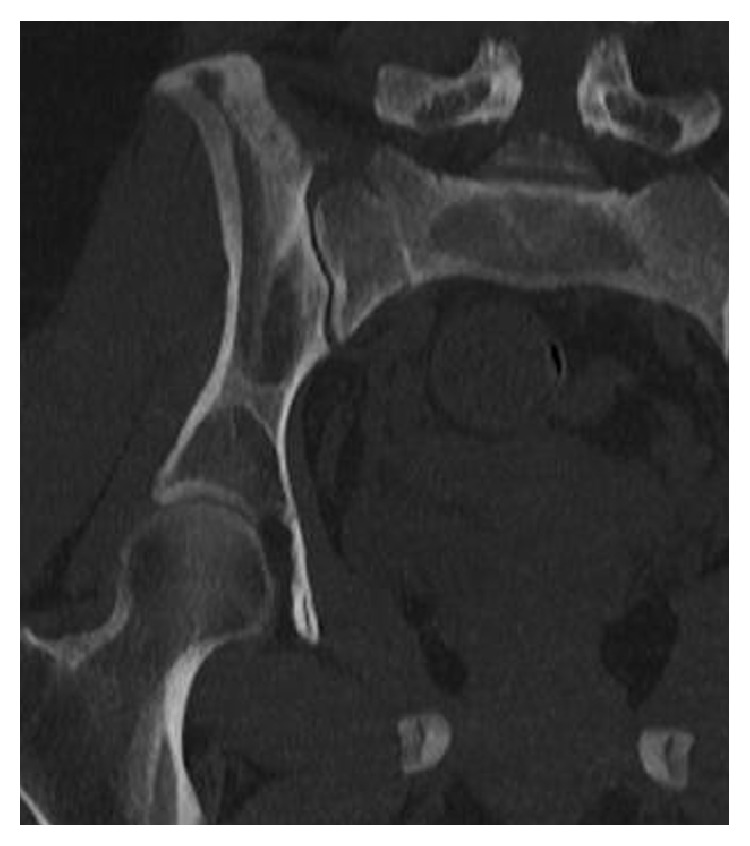
CT scan: coronal view of the right pelvis that showed the fracture line from the superior to the inferior border of the sacral bone with disruption of the cortical bone.

**Figure 3:**
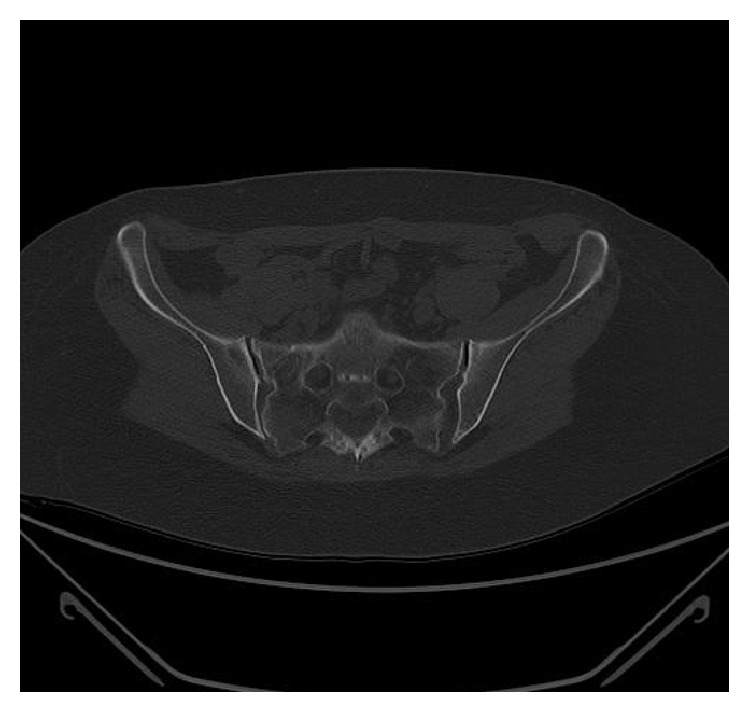
CT scan: axial view that showed the fracture line from the anterior border of sacrum to the right sacroiliac joint.

**Figure 4:**
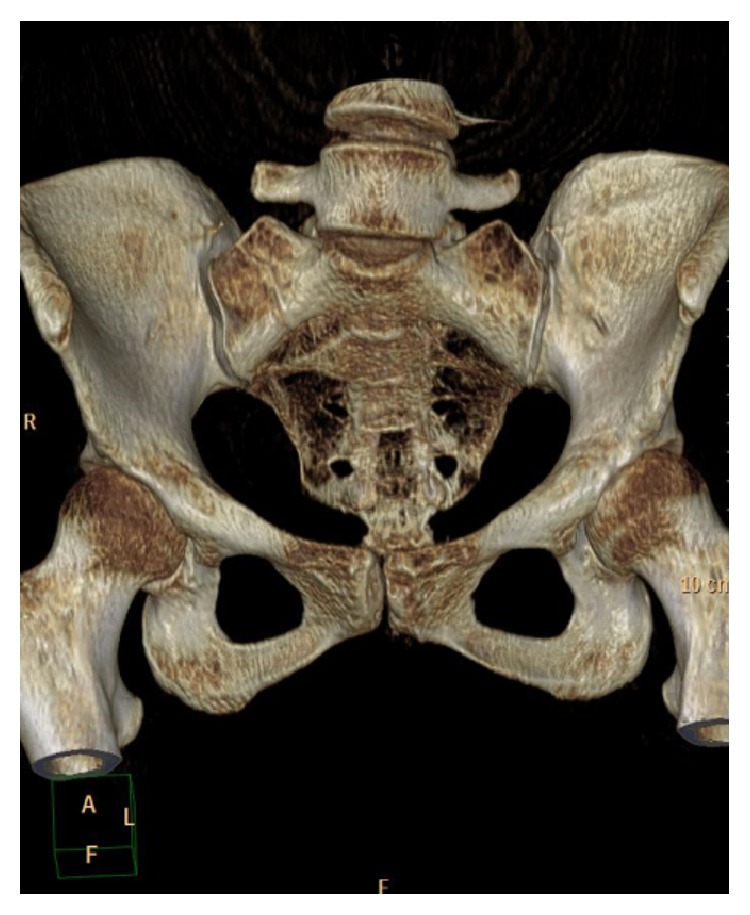
CT scan 3D reconstruction of the pelvis that showed the oblique pattern of fracture line of right sacral bone.

Radiographic views were negative for any fractures. Then, the patient performed MRI investigation that excluded transient osteoporosis of the hip and showed bone marrow edema at the sacrum (Figure 1). Finally, the CT scan confirmed the fracture of the sacrum (Figures 2 and 3). In addition, a 3D reconstruction was performed using CT scans (Figure 4).

Serum level of calcium, phosphorus and alkaline phosphatase, thyroid and parathyroid hormones, serum 25-(OH) vitamin D_3_, and urinary excretion of calcium were normal.

Acetaminophen was used for pain control. Partial weight bearing was allowed for 6 weeks; then she had complete pain relief by 8 weeks.

## 3. Discussion

Sacral stress fracture is uncommon in young population; however, it should be considered during pregnancy and the postpartum period. To the best of our knowledge, only nine cases of postpartum sacral fractures have been reported in the literature [2, 4–11]. Pishnamaz and colleagues [12] reported a case of sacral fracture during pregnancy. Only J. Narvaez and J. A. Narvaez [10] described another case of postdelivery sacral fracture after cesarean section.

Only six cases have reported the patient's bone density [5–7, 9–11]; of them only one showed decreased bone density [5]. Our patient had no risk factors for osteoporosis except for pregnancy and lactation.

Contribution of mechanical factors during the delivery is not a reasonable cause of sacral fracture, because the cesarean cannot be responsible either for the fracture or for the pain.

In 1964, Pentecost and colleagues described two types of fractures [13]: fatigue fracture due to abnormal and repetitive stress and insufficiency fracture in a weakened bone under normal stress.

Fatigue fracture is the most likely diagnosis in our patient, due to stress related to rapid and excessive weight gain in the last trimester of pregnancy.

Focal osteoporosis indicates a thinner thumbnail-sized patch of cortical bone which needs CT scans to be visible [14].

Transient osteoporosis also referred to as Bone Marrow Edema Syndrome affects women in their last trimester of pregnancy, especially involving femoral head and neck [15].

Although it is challenging to assess, transient and/or focal osteoporosis of the sacrum correlated with pregnancy and lactation may have been responsible for the fracture.

Usually sacral stress fracture occurs with low back pain and buttock tenderness but radicular symptoms have been reported by Lin and Lutz [11] and Aylwin et al. [16].

Physical examination can rule out other causes of low back pain and buttock tenderness; however, imaging is helpful in differential diagnoses [17].

In our case the diagnosis was delayed for two days probably due to the lack of specificity of the symptoms.

Increased levels of relaxin, excessive weight gain, hyperlordosis, weakness of the pelvic ligaments, osteopenia caused by increased level of prolactin, and osteoporosis of pregnancy may contribute to the development of sacral fractures [8].

Pregnancy and lactation could be risk factors for sacral stress fracture even in atraumatic delivery such as cesarean section.

Literature data showed that only 16.6% of patients reported decreased mineral bone density.

Osteoporosis associated with pregnancy as a possible cause for sacral fracture was first described by Nordin and Roper [18]. The pathogenesis is unknown and it usually appears during the third trimester or shortly after delivery [19, 20].

In accordance with other authors [2] who state that the fracture can be neglected until 3 weeks after the onset of symptoms, X-ray scan was negative.

MRI showed better sensitivity to detect the fracture: it especially showed bone marrow edema that is expression of acute or subacute fracture [21].

CT scans confirmed and better detected the fracture line. We achieved a complete relief of symptoms by 8 weeks. Partial weight bearing as tolerated was allowed for 6 weeks.

Weight bearing could dislocate the fracture line; however, the risk of bony displacement is very low and bearing could enhance healing process of the fracture and it seems necessary to stimulate osteoblastic activity [2, 11]. Other authors described early mobilization and partial weight bearing as tolerated [22].

We have demonstrated that women during the postpartum period can be at high risk of sacral fracture, even in case of atraumatic delivery such as cesarean, and it should be always considered in differential diagnosis of low back pain.
